# Rethinking Conservation in the Anthropocene—The Case of Holobionts

**DOI:** 10.53941/dbgs.2025.100002

**Published:** 2024-10-11

**Authors:** Seyed E. Hasnain, Niyaz Ahmed

**Affiliations:** 1 Department of Biochemical Engineering and Biotechnology, Indian Institute of Technology, Delhi (IITD), New Delhi 110016, India; 2 Department of Life Science, School of Basic Sciences and Research, Sharda University, Greater Noida 201306, India; 3 Department of Biotechnology and Bioinformatics, University of Hyderabad, Hyderabad 500046, India

Eukaryotic organisms are rarely independent entities and very often complex assemblages of hosts and microbial communities, part of an inter-dependent ‘meta-organism’ [1], collectively referred to as holobionts [2]. The relatively new concept of holobionts encompasses a ‘host’ and all its associated microorganisms and all of their collective genomes form the ‘hologenome’ [3,4]. All constituents of a holobiont share niches within the host’s biological system. These interdependent relationships, involving hosts and their associated microorganisms play a crucial role in ecological and evolutionary dynamics. From an evolutionary standpoint, holobionts challenge the neo-Darwinian focus on the individual organism. Consequently, the survival of species depends on the coevolution of the host and its microbiota, with rapid microbial evolution allowing hosts to adapt quickly to environmental changes. However, the loss of biodiversity in the Anthropocene [5] might lead to loss of habitat and or the collapse of the holobionts or their ecosystems. Therefore, the conservation ideas should apply to the whole of the ‘meta-organism’ rather than only to the host species or in a species specific manner [6].

## Symbiotic relationships in a holobiont

It is a well-established concept that microbiota plays a pivotal role in maintaining host’s health, shaping both its ecological and evolutionary dynamics by sustaining vital interrelationships [7]. As a result, any perturbation of the host-microbiota equilibrium may lead to shift from healthy to pre-disease to disease states, and vice-versa [8,9]. Hence, when studying host-microbe interactions, the concept of the holobiont provides a more comprehensive and holistic approach to understand the host in parallel with its associated microorganisms. It is also important to consider microbe-microbe interactions within the holobionts by way of a framework of multipartner, diffuse coevolution [9-11]. Hologenomes offer an enormous opportunity to learn distributed networks and hierarchies of genome encoded web of functions that often criss-cross in a seamless manner. Integrative interaction between the genomes of microorganisms and their hosts facilitates multipartite adaptations and development of metabolism, physiology and behavior ultimately contributing to the selection of a ‘superorganism’ (holobiont). In extended evolutionary time-scale, the frequent interactions between members of the holobiont facilitate the exchange of genetic materials via horizontal gene transfer (HGT) among themselves [9].

The constituent members of a holobiont interact in various symbiotic relationships—ranging from mutualism to parasitism. Microbes contribute to the host’s metabolism, physiology, and behavior, influencing nutrient cycles and immune functions. Coral reefs, for instance, are prime examples of holobionts, where symbiosis between corals and microbes is vital for the reef’s survival [12-14]. In addition to host-microbe dynamics, microbe-microbe interactions within the holobiont significantly influence overall health. These relationships may evolve through HGT, allowing genetic material to move between microbial species, enhancing adaptability. With growing anthropogenic pressures, the concept of holobionts offers a comprehensive model system and an opportunity to explore the impacts of human activity on biodiversity and ecosystem health, particularly in the context of changing urban habitats wherein the communities are jostling with non-communicable diseases and various other chronic ailments [15,16].

## Anthropogenic threats to holobionts

Anthropogenic activities significantly disrupt the delicate balance between the host and its associated microbiota, impacting the overall health of holobionts. This disruption affects both the host and microbial communities, leading to weakened immunity and higher susceptibility to diseases. For example, ocean acidification can destabilize the symbiotic relationships between marine organisms and their microbes, leading to population decline [17]. Antibiotic misuse disrupts the balance of microbial communities in humans and animals, leading to antimicrobial-resistant bacteria which outcompete the microbial communities within holobionts and cause dysbiotic ecosystems and adverse health outcomes. Biodiversity loss, particularly in areas with low species diversity, increases the vulnerability of hosts to pathogenic microbes.

Studies indicate that individuals exposed to environments with greater biodiversity are generally less susceptible to diseases regardless of their socioeconomic status, compared to those living in areas with lower biodiversity [15]. Additionally, factors such as climate change, industrialization, and population migration are reshaping microbial biodiversity, often introducing pathogenic strains to new regions and raising the risk of infections. Given these complexities, studying the holobionts as an integrated mega organism, rather than focusing separately on the host, microbiota, and their intimate environment and niches, is essential for understanding the broader impacts of anthropogenic influences, particularly within the One Health framework.

## Conservation implications

In recognizing holobionts as fundamental ecological units, it is to be emphasized that the conservation strategies must expand beyond the traditional species-centric focus [6]. Holobiont conservation integrates the host and its microbiota, considering the intricate relationships that sustain life. To preserve biodiversity in the face of anthropogenic pressures, holistic conservation efforts should include:

1. Habitat Protection: Ecosystem protection is essential to maintaining the microbial relationships within holobionts. For instance, safeguarding coral reefs helps maintain their delicate symbiotic partnerships [14].
2. Climate Change Mitigation: Addressing global warming [18] can reduce environmental stressors that threaten the survival of holobionts.
3. Microbiome Research: Studying the microbial communities associated with endangered species helps identify critical factors for their survival. Restoring microbial diversity in degraded ecosystems can enhance the resilience of holobionts [6,19].

Recent advances and narratives propose conservation strategies based on microbiome and ecosystem restoration, and hybrid or interdisciplinary approaches [5,6,12,13,20]. Such approaches hold promise to restore or enhance microbial diversity and ecosystem health in degraded habitats also through enhancing the intrinsic capacities of the ecosystems and holobionts. However, one may like to ascertain the fidelity of association among genetic variants of host and microbes to know if they actually co-evolve and co-adapt as a unit [20]. By understanding and manipulating microbial communities together with their host, we can improve the health and survival of endangered species as holobionts. Probiotic treatments, dietary modifications, and ecosystem restoration efforts, including microbial inoculation, are being explored to maintain the integrity of holobiont systems. Holobionts are also critical in ecosystem functions such as nutrient cycling and energy flow, making their preservation essential for maintaining ecological balance.

The holobiont concept offers unique opportunities for public engagement in conservation efforts. Educating the public about the interconnectedness of life and the importance of microbial diversity fosters a greater appreciation and care for nature and encourages individual and collective action towards conservation. Antibiotic stewardship targeting responsible production and use of antimicrobials needs a lot of outreach and advocacy, also in the context of public health, urban ecosystems, sustainable farming and collective wellness. The holobiont and hologenome paradigms present a new frontier in conservation science. By understanding organisms as complex systems of hosts and microorganisms, we can develop more effective strategies [13,14] for protecting biodiversity. As human-induced environmental changes accelerate, adopting a holistic approach to conservation—one that entails entire holobionts —is paramount to the future of life on Earth. Needless to iterate, the importance of such an approach to overall health and well-being in the wider context of One Health cannot be overstated.

## References

[1] Bosch TC; McFall-Ngai MJ Metaorganisms as the new frontier. Zoology 2011, 114, 185–190. 21737250 10.1016/j.zool.2011.04.001 PMC3992624

[2] Margulis L; Fester R Symbiosis as a Source of Evolutionary Innovation: Speciation and Morphogenesis; MIT Press: Cambridge, MA, USA, 1991. 11538111

[3] Zilber-Rosenberg I; Rosenberg E Role of microorganisms in the evolution of animals and plants: The hologenome theory of evolution. FEMS Microbiol. Rev 2008, 32, 723–735. 18549407 10.1111/j.1574-6976.2008.00123.x

[4] Moran NA; Sloan DB The hologenome concept: Helpful or hollow? PLoS Biol. 2015, 13, e1002311. 26636661 10.1371/journal.pbio.1002311 PMC4670207

[5] Miraldo A; Li S; Borregaard MK; Flórez-Rodríguez A; Gopalakrishnan S; Rizvanovic M; Wang Z; Rahbek C; Marske KA; Nogués-Bravo D An Anthropocene map of genetic diversity. Science 2016, 353, 1532–1535. 27708102 10.1126/science.aaf4381

[6] Limborg MT; Winther-Have CS; Morueta-Holme N; Gilbert MTP; Rasmussen JA The overlooked biodiversity loss. Trends Ecol. Evol 2024, 39, 889–891. 39209587 10.1016/j.tree.2024.08.001

[7] Singh Y; Hasnain SE Holobionts: Emerging strategy for interventions against infectious diseases, metabolic disorders & cancer. Indian J. Med. Res 2014, 140, 11–14. 25222772 PMC4181143

[8] Amarasekare P; Possingham H Patch dynamics and metapopulation theory: The case of successional species. J. Theor. Biol 2001, 209, 333–344. 11312593 10.1006/jtbi.2001.2269

[9] Simon JC; Marchesi JR; Mougel C; Selosse MA Host-microbiota interactions: From holobiont theory to analysis. Microbiome 2019, 7, 5. 30635058 10.1186/s40168-019-0619-4 PMC6330386

[10] Singh Y; Ahmad J; Musarrat J; Ehtesham NZ; Hasnain SE Emerging importance of holobionts in evolution and in probiotics. Gut Pathog. 2013, 5, 1–8. 23694677 10.1186/1757-4749-5-12 PMC3668144

[11] Richardson LA Evolving as a holobiont. PLoS Biol. 2017, 15, e2002168. 28245221 10.1371/journal.pbio.2002168 PMC5330447

[12] Bourne DG; Morrow KM; Webster NS Insights into the coral microbiome: Underpinning the health and resilience of reef ecosystems. Annu. Rev. Microbiol 2016, 70, 317–340. 27482741 10.1146/annurev-micro-102215-095440

[13] van Oppen MJ; Blackall LL Coral microbiome dynamics, functions and design in a changing world. Nat. Rev. Microbiol 2019, 17, 557–567. 31263246 10.1038/s41579-019-0223-4

[14] van Oppen MJ; Oliver JK; Putnam HM; Gates RD Building coral reef resilience through assisted evolution. Proc. Natl. Acad. Sci 2015, 112, 2307–2313. 25646461 10.1073/pnas.1422301112 PMC4345611

[15] Mills JG; Brookes JD; Gellie NJ; Liddicoat C; Lowe AJ; Sydnor HR; Thomas T; Weinstein P; Weyrich LS; Breed MF Relating urban biodiversity to human health with the ‘holobiont’ concept. Front. Microbiol 2019, 10, 550. 30972043 10.3389/fmicb.2019.00550 PMC6444116

[16] Blaser MJ The theory of disappearing microbiota and the epidemics of chronic diseases. Nat. Rev. Immunol 2017, 17, 461–463. 28749457 10.1038/nri.2017.77

[17] Hoegh-Guldberg O; Poloczanska ES; Skirving W; Dove S Coral reef ecosystems under climate change and ocean acidification. Front. Mar. Sci 2017, 4, 158.

[18] Hughes TP; Kerry JT; Álvarez-Noriega M; Álvarez-Romero JG; Anderson KD; Baird AH; Babcock RC; Beger M; Bellwood DR; Berkelmans R; Global warming and recurrent mass bleaching of corals. Nature 2017, 543, 373–377. 28300113 10.1038/nature21707

[19] Rosenberg E; Zilber-Rosenberg I Microbes drive evolution of animals and plants: The hologenome concept. mBio 2016, 7, 10–1128. 10.1128/mBio.01395-15 PMC4817260 27034283

[20] Douglas AE; Werren JH Holes in the hologenome: Why host-microbe symbioses are not holobionts. mBio 2016, 7, e02099–15. 27034285 10.1128/mBio.02099-15 PMC4817262

